# Genomic sequencing of 10 spore-forming *Bacilli* strains isolated from zoo-dwelling mice

**DOI:** 10.1128/mra.00446-24

**Published:** 2024-07-31

**Authors:** Rachel Hashuel, Eran Gutman, Yael Litvak

**Affiliations:** 1 Department of Biological Chemistry, The Alexander Silberman Institute of Life Sciences, The Hebrew University of Jerusalem, Edmond J. Safra Campus Givat-Ram, Jerusalem, Israel; Portland State University, Portland, Oregon, USA

**Keywords:** Bacillus, endospores, gut microbiome, soil microbiology

## Abstract

We present the genomic sequences of 10 spore-forming bacteria from the *Bacillaceae* family isolated from fecal samples of mice residing in the Tisch Family Biblical Zoo, Jerusalem. These isolates suggest Bacillus bacteria are a native component of rodent gut flora, facilitating further research into gut colonization and microbiome diversity.

## ANNOUNCEMENT

The vast microbial landscape inhabiting the animal gut, known as the microbiome, plays a vital role in digestion and overall health (1). Environmental factors like diet and habitat significantly shape its composition (2–4). Rodents housed in a city Zoo are exposed to a broad spectrum of microbes from various animal species, providing an opportunity to explore gut microbiome diversity. Our study focused on the isolation and characterization of spore-forming *Bacilli* bacteria from fresh feces of mice in the Tisch Family Biblical Zoo in Jerusalem, Israel. These robust microorganisms, renowned for their resistant spores that can withstand extreme temperatures and harsh environmental conditions, are primarily known as inhabitants of soil (5). However, our work reveals their surprising prevalence and potential adaptations within the mammalian gut, opening avenues for studying their contribution to host health and potentially novel functions within this niche.

### Provenance and isolation

In July 2020, fresh feces from zoo-dwelling mice, used for reptile feeding and not for exhibition, were collected (sample collection coordinates: 31.748047 and 35.177728). Animals were observed, and when fresh feces were produced, the content was carefully sampled using sterile instruments, avoiding any material that had come into contact with the soil. The content was homogenized with 1 mL of phosphate-buffered saline and was subjected to 65°C heat for 60 minutes to eliminate vegetative cells and to select for spores (6). Two hundred microliter of the heat-treated sample was then plated onto Lysogeny broth (LB) agar (7) and incubated at 37°C for 18 hours, allowing the spores to germinate and form colonies. Colonies were re-streaked onto fresh LB agar to ensure purity and also onto MacConkey plates, which are selective for Gram-negative bacteria, to confirm the Gram-positive nature of the colonies (8, 9). Single isolated colonies were cultured in liquid LB media at 37°C, and genomic DNA was extracted from a 2 mL overnight culture using the Wizard kit (Promega, Madison, WI) according to the manufacturer’s instructions.

Genomic DNA was sequenced by MicrobesNG (Birmingham, UK) using an Illumina NovaSeq 6000 instrument and following their standard workflow for library preparation and read trimming. This workflow uses the Nextera XT Library Prep Kit (Illumina, San Diego, USA) following the manufacturer’s protocol with the following modifications: input DNA was increased twofold, and PCR elongation time was increased to 45 seconds. DNA quantification and library preparation were carried out on a Hamilton Microlab STAR automated liquid handling system (Hamilton Bonaduz AG, Switzerland). Libraries were sequenced using a 250 bp paired-end protocol. Reads were adapter trimmed using Trimmomatic version 0.30 (10) with a sliding window quality cutoff of Q15. Read quality was assessed using MicrobesNG in-house scripts combined with Samtools, BedTools, and bwa-mem software (11–13). *De novo* assembly was performed on samples using SPAdes version 3.14.1, and contigs are annotated using PGAP version 6.5 (14, 15). All tools were run with default parameters unless otherwise specified. Table 1 lists the genome assembly statistics, number of reads, and other parameters for the 10 *Bacilli* strains. Taxonomic labels were assigned using Kraken v1 (16).

**TABLE 1:** Summary of genomic features

Strain	Isolate identifier	Total length (bp)	No. of contigs	GC content (%)	Number of coding sequences	Mean coverage	Number of reads	N50	Number of tRNA	Assembly	GenBank WGS accession no.	SRA accession no.
*Priestia megaterium*	RU281	6,212,340	112	37.4	6,406	191.5	2,507,337	4,212,224	126	GCA_030676525.1	JAUUTN000000000	SRR27759687
*P. megaterium*	RU282	5,760,607	63	37.5	5,968	213.8	2,553,692	4,126,778	126	GCA_030676555.1	JAUUTO000000000	SRR27759688
*Peribacillus simplex*	RU283	5,788,502	220	39.5	5,666	137.2	1,671,331	125,747	84	GCA_030676475.1	JAUUTP000000000	SRR27759689
*Lysinibacillus capsici*	RU284	4,653,943	60	37.5	4,593	168.2	1,633,080	410,664	85	GCA_030676485.1	JAUUTQ000000000	SRR27759690
*L. capsici*	RU285	4,654,572	59	37.5	4,615	214.8	2,102,191	410,664	84	GCA_030676425.1	JAUUTR000000000	SRR27759691
*P. megaterium*	RU286	5,592,861	79	37.5	5,765	187.5	2,185,810	4,126,778	124	GCA_030676395.1	JAUUTS000000000	SRR27759692
*P. megaterium*	RU287	6,087,749	105	37.5	6,264	103.1	1,314,731	4,212,224	127	GCA_030676515.1	JAUUTT000000000	SRR27759693
*L. capsici*	RU291	4,651,476	53	37.5	4,605	99.1	1,085,146	410,664	86	GCA_030676375.1	JAUUTU000000000	SRR27759694
*Bacillus wiedmannii*	RU292	5,365,704	204	35.5	5,534	180.2	2,077,025	104,638	96	GCA_030676405.1	JAUUTV000000000	SRR27759695
*Peribacillus frigoritolerans*	RU293	5,787,954	190	39.5	5,659	175.8	2,114,695	130,873	83	GCA_030676415.1	JAUUTW000000000	SRR27759696

## Data Availability

The standardized strain descriptions and accession numbers are presented in Table 1; the genomic data are publicly available in GenBank under BioProject no. PRJNA1000183.
